# Modulation of the rat hippocampal‐cortex network and episodic‐like memory performance following entorhinal cortex stimulation

**DOI:** 10.1111/cns.13795

**Published:** 2021-12-28

**Authors:** Yin Jiang, De‐Feng Liu, Xin Zhang, Huan‐Guang Liu, Chao Zhang, Jian‐Guo Zhang

**Affiliations:** ^1^ Department of Functional Neurosurgery Beijing Neurosurgical Institute Capital Medical University Beijing China; ^2^ Beijing Key Laboratory of Neurostimulation Beijing China; ^3^ Department of Functional Neurosurgery Beijing Tiantan Hospital Capital Medical University Beijing China

**Keywords:** deep brain stimulation, entorhinal cortex, episodic‐like memory, functional connectivity, hippocampal‐cortex network

## Abstract

**Aims:**

Entorhinal cortex (EC) deep brain stimulation (DBS) has shown a memory enhancement effect. However, its brain network modulation mechanisms remain unclear. The present study aimed to investigate the functional connectivity in the rat hippocampal‐cortex network and episodic‐like memory performance following EC‐DBS.

**Methods:**

7.0 T functional MRI (fMRI) scans and episodic‐like memory tests were performed 3 days and 28 days after EC‐DBS in healthy rats. The fMRI data processing was focused on the power spectra, functional connectivity, and causality relationships in the hippocampal‐cortex network. In addition, the exploration ratio for each object and the discrimination ratio of the “when” and “where” factors were calculated in the behavioral tests.

**Results:**

EC‐DBS increased the power spectra and the functional connectivity in the prefrontal‐ and hippocampal‐related networks 3 days after stimulation and recovered 4 weeks later. Both networks exhibited a strengthened connection with the EC after EC‐DBS. Further seed‐based functional connectivity comparisons showed increased connectivity among the prefrontal cortex, hippocampus and EC, especially on the ipsilateral side of DBS. The dentate gyrus is a hub region closely related to both the EC and the prefrontal cortex and receives information flow from both. Moreover, acute EC‐DBS also enhanced the discrimination ratio of the “where” factor in the episodic‐like memory test on Day 3.

**Conclusion:**

EC‐DBS caused a reversible modulation effect on functional connectivity in the hippocampal‐cortex network and episodic‐like memory performance.

## Graphical abstract

Entorhinal cortex (EC) deep brain stimulation (DBS) increased the power spectra and the functional connectivity in the prefrontal cortex‐ and hippocampus‐related networks 3 days after stimulation and recovered 4 weeks later, accompanied by enhanced discrimination ratio of the “where” factor in the episodic‐like memory test. In conclusion, EC‐DBS caused a reversible modulation effect on functional connectivity in the hippocampal‐cortex network and episodic‐like memory performance.

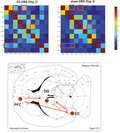

## 1 INTRODUCTION

Increasing preclinical studies and clinical trials have shown that deep brain stimulation (DBS) has potential for treating dementia‐related disorders.^1^, ^2^, ^3^, ^4^, ^5^, ^6^ Among these studies, the entorhinal cortex (EC)‐DBS has shown a memory enhancement effect both in patient and animal studies. Suthana et al. implanted intracranial depth electrodes in epilepsy patients and revealed that EC stimulation enhanced memory of spatial information.^7^ Improved episodic memory performance was also reported in a recent study using the macrostimulation method to deliver EC‐DBS in neurosurgical patients.^8^ In addition, animal studies from our team and other researchers also demonstrated improved behaviors in memory‐impaired rats after EC‐DBS.^9^, ^10^, ^11^, ^12^, ^13^

To date, research exploring the neuromodulation mechanism of EC‐DBS has been limited. Previous animal studies reported that acute EC‐DBS promoted neurogenesis in the dentate gyrus (DG) 3–5 days after stimulation. Once sufficiently mature, these neurons integrated into hippocampal circuits supporting spatial memory.^14^ These studies suggest that EC‐DBS‐induced memory enhancement is closely related with the hippocampus, and there is a process of modulation from short‐term to long‐term. It is worth noting that neurogenesis‐related memory improvements were mostly observed several weeks after EC‐DBS; however, in the studies of patients, EC‐DBS exhibited an acute enhancement effect on memory performance. These conflicting may be due to the different types of behavioral tests in human (episodic memory) and animal (spatial memory) studies. Moreover, it is also reasonable to infer that in addition to DG neurogenesis, EC‐DBS could have other short‐term neuromodulation mechanisms of regulating memory function.

Multiple functional MRI (fMRI) studies showed that altered resting‐state functional connectivity of the hippocampal‐cortex network is associated with the severity of memory decline in both cognitive impairment patients and animals.^15^, ^16^, ^17^, ^18^ The EC‐hippocampus interaction is generally accepted to play an important role in memory processing.^19^, ^20^ Previous studies revealed that the impact on memory encoding‐related potentials within the hippocampus could result from EC stimulation in neurosurgical patients.^7^, ^21^ In addition, an increasing number of studies have demonstrated that memory consolidation involves strengthening the connection between the EC and the prefrontal cortex rather than the hippocampus.^22^, ^23^ Recently, Krautwald et al. further reported a stronger response in the prefrontal cortex (PFC) compared with the hippocampus during EC stimulation, indicating that the acute beneficial effects of EC‐DBS may depend on both prefrontal and hippocampal functions.^24^ However, to our knowledge, EC‐DBS‐induced functional connectivity changes in the hippocampal‐cortex network, including the prefrontal cortex, hippocampus, and EC, remain unclear.

The objective of the present study was to observe the functional hippocampal‐cortex network changes after acute EC‐DBS in the rat brain using 7.0 T fMRI. Short‐term and long‐term effects were studied three days and twenty‐eight days after stimulation, corresponding to the time points of acute neurogenesis and long‐term memory improvement caused by EC‐DBS in previous animal studies,^9^, ^14^ respectively. Data analysis was focused on prefrontal‐ and hippocampal‐related networks. In addition, an episodic‐like memory test was also performed at different time points.^25^ We hypothesize that the resting‐state hippocampal‐cortex functional connectivity and episodic‐like memory performance could be modulated by acute EC‐DBS.

## 2 METHODS

### 2.1 Animals

Thirty‐six 6‐week‐old male Sprague‐Dawley rats (190–210 g, Charles River, China) were housed in groups of two or three per cage under the controlled laboratory conditions (12 h light/dark cycle with the ambient temperature of 22 ± 2°C). Food and water were provided *ad libitum*. All procedures complied with the ARRIVE guidelines and were performed following the U.K. Animals (Scientific Procedures) Act, 1986. Protocols were approved by the animal ethics committee of Beijing Neurosurgical Institute.

### 2.2 Electrical stimulation of the EC

**FIGURE 1:**
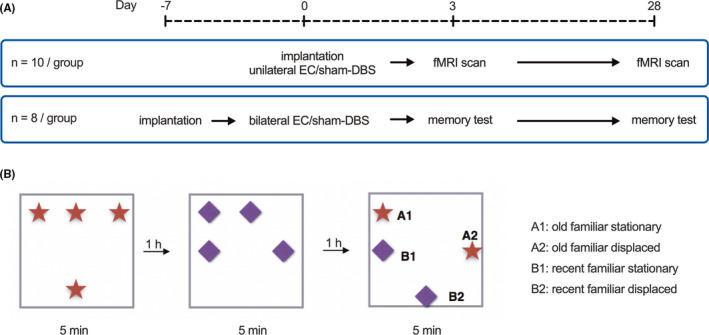
(A) Schematic illustration of the experimental timeline. (B) Details of episodic‐like memory test. After animals explored four identical objects on the first session and four other identical objects on the second session, the exploratory time of rats on the four objects (A1, old familiar stationary; A2, old familiar displaced; B1, recent familiar stationary; B2, recent familiar displaced) was recorded on the test session

Animals were anesthetized with isoflurane and placed in a stereotactic frame (Kopf, Germany). Concentric bipolar electrodes (FHC, Bowdoin, ME, USA) were implanted into the EC according to coordinates in the Paxinos and Watson rat brain atlas (AP: −6.7 mm; ML: ±4 mm; DV: −8 mm).^26^ Twenty animals received unilateral implantation in the left EC. Rats were randomly assigned to the EC‐DBS group or sham‐DBS group. In the EC‐DBS group, stimulation was delivered using a pulse stimulator (Master 8, AMPI, Israel) for 1 h at parameters that were the same as those in our previous report (130 Hz, 90 µs, and 500 µA).^9^ Animals in the sham‐DBS group only had electrode implantation but did not receive stimulation. Following stimulation, the electrode was removed, the surgical incision was closed, and the animals were allowed to recover. These twenty animals received fMRI scans 3 days and 28 days after stimulation. T2‐weighted MRI were used to determine the location of the electrode target area, and the localization of the electrode tip in the left EC is illustrated in Figure S1a. Other sixteen animals received bilateral EC implantation. To avoid influence of postoperative cognitive dysfunction, animals were given a one‐week recovery period after the electrodes were fixed with dental cement. Animals were then randomly assigned to the EC‐DBS and sham‐DBS group, and bilateral stimulation parameters were the same as the unilateral stimulation. These sixteen rats received episodic‐like memory tests 3 days and 28 days after stimulation. Histological staining was used to confirm the accuracy of the bilateral coordinate of EC (Figure S1b). The Schematic illustration of the experimental grouping and timeline were showed in Figure 1a.

### 2.3 functional MRI data acquisition and preprocessing

Twenty animals (*n* = 10/group) underwent two fMRI scan sessions 3 days and 28 days after stimulation. All MR images were acquired using a 7.0 T MRI scanner (BioClinScan, Bruker, Germany). Rats were anesthetized with isoflurane (2%) and secured in a head holder. A physiological monitoring system was used to monitor the respiratory rate and body temperature (BioTrig, Bruker). Functional images were acquired using echo‐planar imaging with the following parameters: TR =2000 ms, TE =15 ms, flip angle =90°, matrix size =64 × 64, FOV =125 mm ×125 mm, slice thickness =1 mm, repetitions =300. In the sham‐DBS group, after the first fMRI scan session, one rat died over the following 12 h, and a visible abnormal cavity was observed in the brain tissue of another rat. Thus, 10 rats in the EC‐DBS group and 8 rats in the sham‐DBS group were used in subsequent fMRI data analyses.

Statistical Parametric Mapping (SPM8) software (Wellcome Department of Cognitive Neurology, London, UK) and MATLAB (MathWorks, Natick, MA) were used for fMRI data preprocessing. A standard protocol was utilized according to previous studies.^18^, ^27^, ^29^ To eliminate the inhomogeneous effects of magnetization, the first 10 volumes of each session were removed, and all volumes were realigned and spatially normalized to a standard template and spatially smoothed using a Gaussian filter (1‐mm full width at half maximum).^30^ Then, data were filtered to reduce the effect of low‐frequency drift and high‐frequency noise by using a bandpass filter (0.01–0.08 Hz).

### 2.4 Prefrontal‐ and hippocampal‐related networks analysis

**FIGURE 2:**
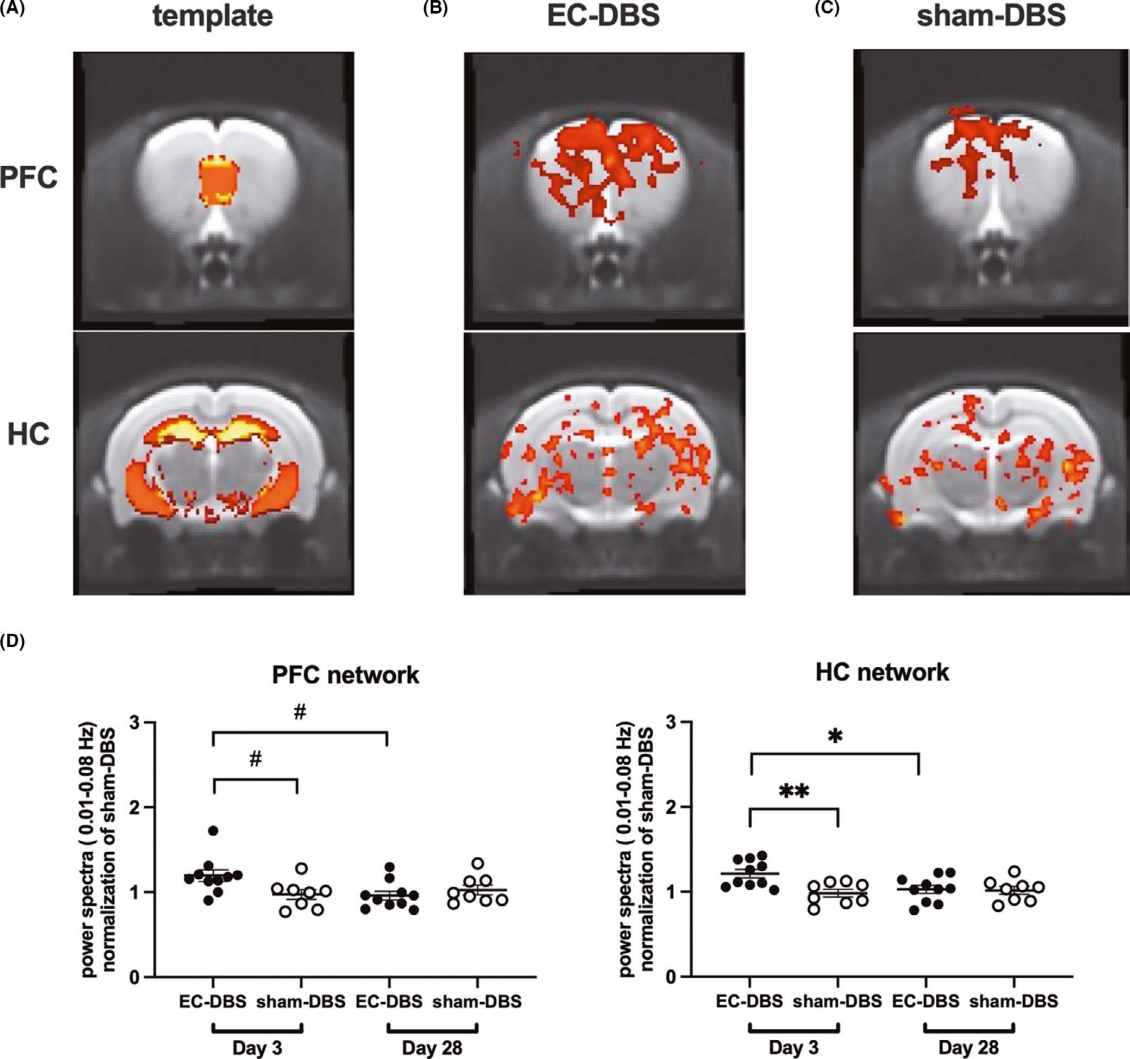
Prefrontal‐ and hippocampal‐related functional networks. (A) The best‐fit components were selected by using the masks of structural PFC and HC. PFC, prefrontal cortex; HC, hippocampus. (B and C) Prefrontal‐ and hippocampal‐related functional network maps of an EC‐DBS and a sham‐DBS rat. D. Power spectra (0.01–0.08 Hz) of the PFC‐ and HC‐related networks in EC‐DBS and sham‐DBS rats 3 days and 28 days after stimulation. Data represented as mean ± S.E. of all points. Multiple Mann‐Whitney tests, #*p *< 0.05; two‐way ANOVA, *p < 0.05, **p < 0.01

To investigate the prefrontal‐ and hippocampal‐related networks, independent component analysis (ICA) was performed using the Group ICA of fMRI Toolbox (GIFT, http://icatb.sourceforge.net/). The number of components was set to 20, as informed by previous rodent studies.^29^, ^31^ The prefrontal‐ and hippocampal‐related networks were identified by spatially sorting all components with the structural prefrontal and hippocampal masks (Figure 2a).^30^ For each subject, the best‐fit component was extracted from each run (Figure 2b and 2c). For each best‐fit component, by using the utility of spectral group comparison in the GIFT, the power spectra were compared between the two groups in two different scan sessions. After the Shapiro‐Wilk test, normally distributed variables were compared by two‐way ANOVA with Bonferroni posttest, while non‐normally distributed variables were compared by multiple Mann‐Whitney tests with FDR correction, and the threshold of significance was set to *p*<0.05. SPM two‐sample *t*‐tests were then performed to determine the differences in the spatial extent of the prefrontal‐ and hippocampal‐related networks between the two groups, and the threshold was set as cluster‐level FDR‐corrected *p *< 0.05.

### 2.5 Seed‐based functional network analysis

In the present study, ten regions of interest (ROIs), including the left and right PFC, DG, CA1, CA3, and EC, were used to perform seed‐based functional connectivity analysis. The center of each seed was located in coronal planes according to the Paxinos and Watson atlas,^26^ and the diameter was set to 1 mm (Figure S2). The time course of blood‐oxygenation‐level‐dependent (BOLD) signals in each session were extracted using REST (http://www.restfmri.net). Afterward, correlations of the signal time courses with the above seeds were calculated. For each rat in each scan session, there was a matrix containing all the correlation rates (r‐value) among the seed regions. The connectivity rate between the EC‐DBS and sham‐DBS groups within each seed‐related connections was compared at different time points, respectively. After the Shapiro‐Wilk test, data were compared by multiple Mann‐Whitney tests with FDR correction, and the threshold of significance was set to *p *< 0.05.

To further observe the causal influence flows changed by EC‐DBS, the abovementioned significantly changed connections were also used for the Granger causality analysis (GCA). ROI‐wise GCA was implemented on REST software (http://www.restfmri.net/forum/REST‐GCA) using multivariate coefficients. For each rat, there was a coefficient between every two seed regions. After the Shapiro‐Wilk test, one sample *t*‐test with Wilcoxon test in GraphPad 6.0 software was used to determine the directional connectivity (coefficient significantly different from zero),^32^, ^33^ with the threshold set at *p *< 0.05.

### 2.6 Episodic‐like memory test and data analysis

Animals received episodic‐like memory tests 3 days or 28 days after bilateral stimulation, and different objects were used on different test days. In the beginning, animals were handled individually on 3 consecutive days and were allowed 10 min free open‐field (50 × 50 × 45 cm) exploration in the empty box for habituation. On the test day, there are two familiarization sessions (training) and one recognition session (testing). During the familiarization, each rat was exposed to four identical objects that were placed in the box for 5 min, and there was an hour intertrial between the two familiarization trials. The recognition trial was performed an hour after the familiarization trial in the same manner for 5 min, except those four objects were placed as old familiar stationary (A1), old familiar displaced (A2), recent familiar stationary (B1), and recent familiar displaced (B2) (Figure 1b). The exploration track was recorded and analyzed off‐line with a computer tracking system (SuperMaze, XinRuan, China). For each trial, the exploration ratio was calculated and normalized (time spent exploring the object/ average of total exploration time of all trials) for each object in each rat. According to previous studies,^25^, ^27^, ^28^ temporal factor (when = [A1 – B1] / [A1 + B1]) and spatial factor (where = [B2 – B1]/ [B2 + B1]) were further calculated based on the exploration ratios and normalized for each trial (/average of all trials). After the Shapiro‐Wilk test, repeated measures one‐way ANOVA with multiple comparison tests was used to compare the exploration ratios within the test. Two‐way ANOVA with multiple comparison tests was used to compare the “when” and “where” factors between the two groups at different time points. The threshold of significance for all the above comparisons was set at *p*<0.05, FDR‐corrected.

## 3 RESULTS

### 3.1 Power spectra changes induced by EC‐DBS

In the present research, by spatially sorting all components with the structural PFC and hippocampus masks, two best matching component maps (one for PFC and one for hippocampus) could be extracted for each scan. The prefrontal‐related network map included the bilateral PFC, orbitofrontal cortex, sensorimotor cortex and insula, whereas the hippocampal‐related network map included the bilateral hippocampus, thalamus, cingulate cortex and somatosensory cortex (Figure 2b and 2c). Further, a power spectra value in 0.01–0.08 Hz could be calculated for each component in each scan. Significant power spectra difference was observed between the EC‐DBS and sham‐DBS groups in both the prefrontal‐ and hippocampal‐related networks. These two networks both exhibited increased low‐frequency power spectra 3 days after EC‐DBS (PFC Mann‐Whitney *U* = 15, *p*<0.05, HC post hoc *t* = 3.38, *p *< 0.01) and recovered to normal levels 28 days after stimulation. (Figure 2d and 2e).

### 3.2 Prefrontal‐ and hippocampal‐related functional networks modulated by EC‐DBS

**FIGURE 3:**
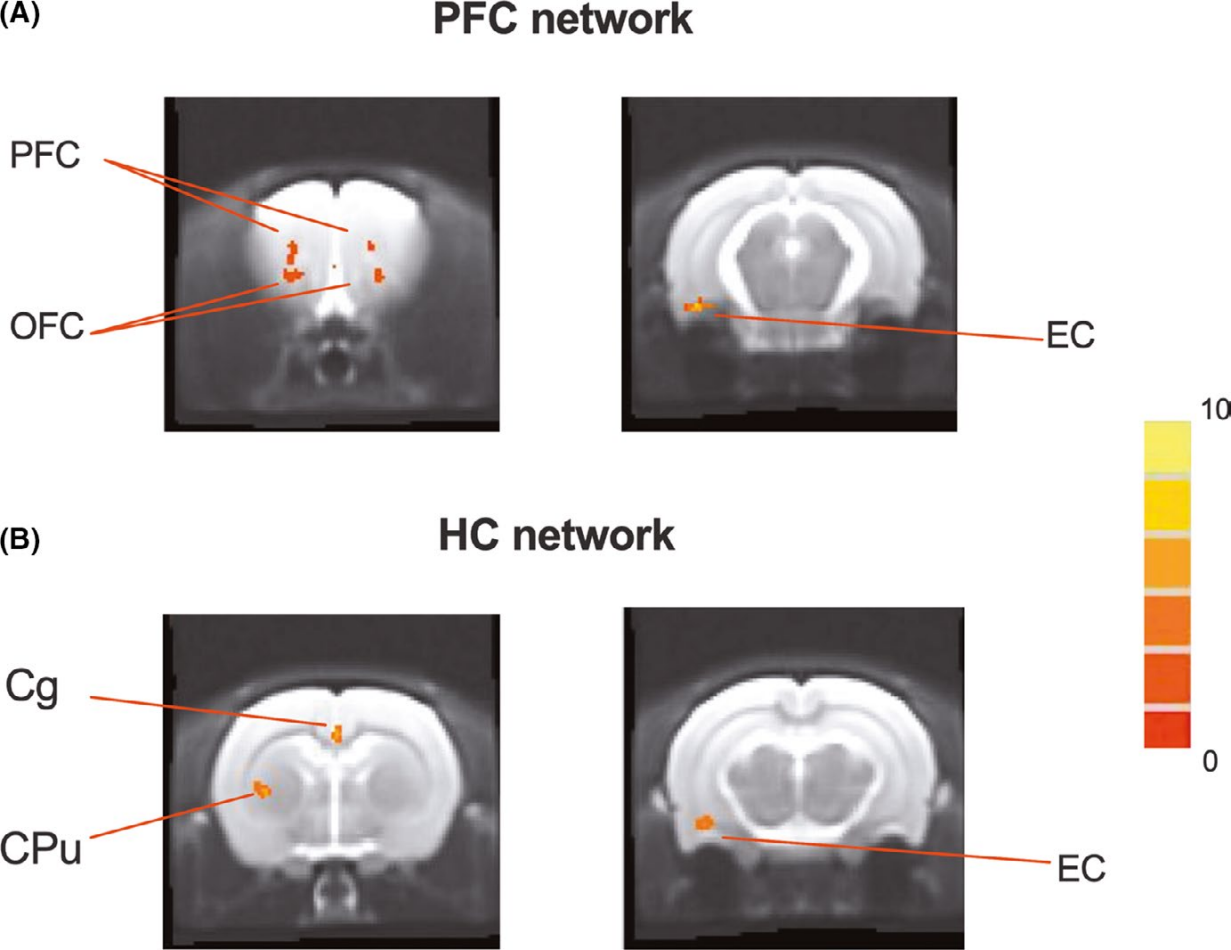
Prefrontal‐ and hippocampal‐related networks changes after EC‐DBS. Brain regions showed increased functional connectivity in prefrontal‐ and hippocampal‐related functional networks in the EC‐DBS group as compared to the sham‐DBS group 3 days after stimulation. Two sample *t*‐test was used in the SPM comparison, and the threshold of display was set to cluster‐level corrected *p*<0.05. The color bar indicates *t* values. PFC, prefrontal cortex; HC, hippocampus; OFC, orbitofrontal cortex; EC, entorhinal cortex; Cg, cingulate cortex; CPu, caudate putamen

Three days after stimulation, compared to sham‐DBS, increased connectivity of the prefrontal‐related network was observed in the bilateral PFC, orbitofrontal cortex and ipsilateral EC after EC‐DBS (Figure 3a). In addition, for the hippocampal‐related network, EC‐DBS also induced increased functional connectivity in the bilateral cingulate cortex, ipsilateral EC and caudate putamen (Figure 3b). There was no spatial extent difference of the prefrontal‐ and hippocampal‐related networks between the two groups on day 28.

### 3.3 Seed‐based functional connectivity and causal influence flow changes after EC‐DBS

**FIGURE 4:**
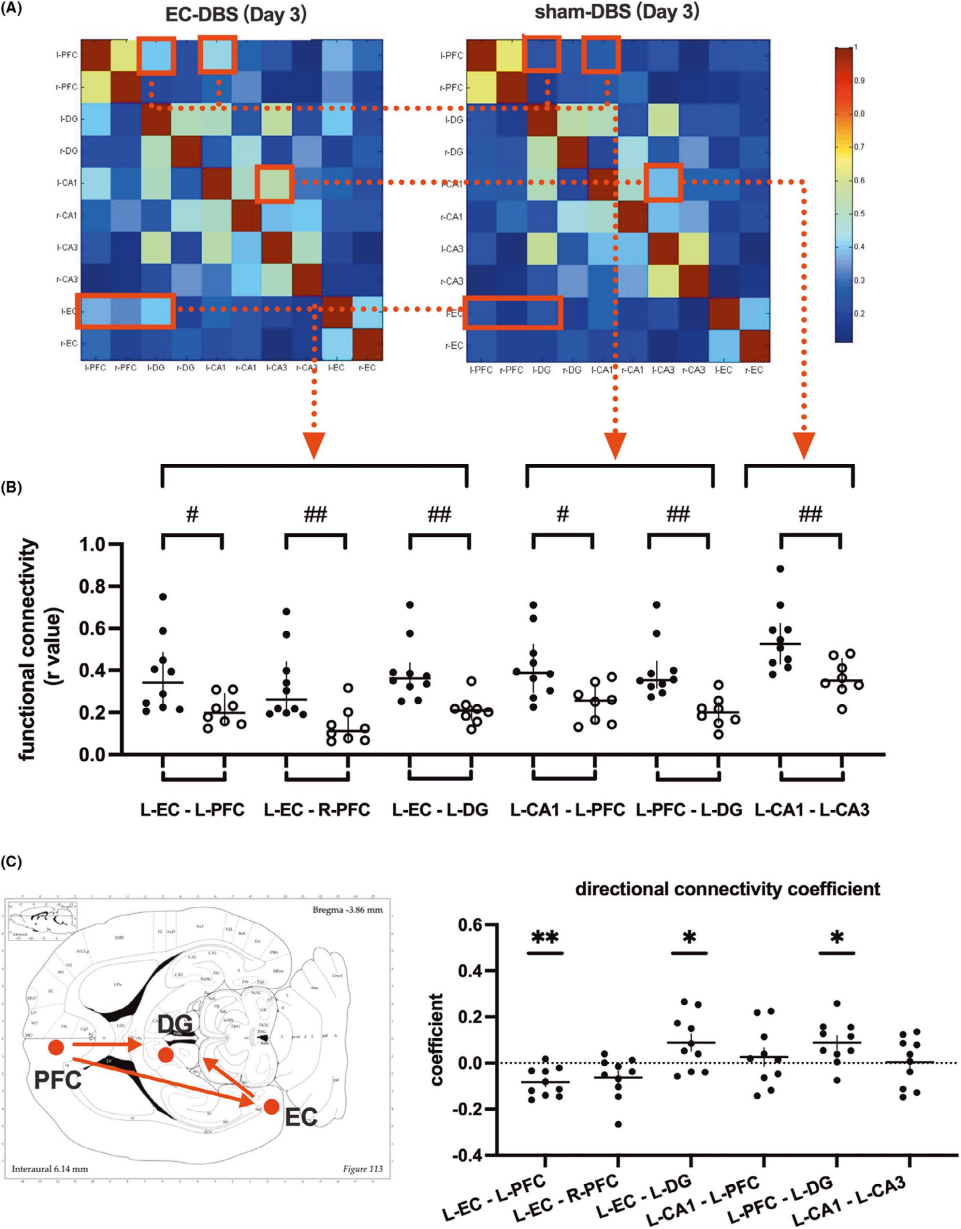
(A) Functional connectivity among brain regions, including the left and right PFC, DG, CA1, CA3 and EC in the EC‐DBS and sham‐DBS group 3 days after stimulation. The color bar indicates mean r‐values. (B) EC‐DBS‐induced functional connectivity changes 3 days after stimulation. Data represented as median with interquartile range of all points. Multiple Mann‐Whitney tests, #*p *< 0.05, ##*p *< 0.01. (C) Granger causality analysis revealed the directions of functional connectivity among the left PFC, DG and EC in EC‐DBS rats 3 days after stimulation. Directional connectivity coefficients of seed‐based functional connections are represented as mean ± S.E. of all points. One sample *t*‐test, *p < 0.05, **p < 0.01

Functional connectivity among ten seed regions, including the left and right PFC, DG, CA1, CA3 and EC, was calculated within each rat, and the mean connectivity values are presented in Figure 4a. Compared to sham‐DBS, increased connectivity between the left EC and the bilateral PFC (L‐EC ‐ L‐PFC U = 17, *p*<0.05; L‐EC – R‐PFC U = 9, *p *< 0.01), the left EC and the left DG (U = 4, *p *< 0.01), the left PFC and the left CA1(U = 7, *p *< 0.05), the left PFC and the left DG (U = 3, *p *< 0.01), and the left CA1 and the left CA3 (U = 3, *p*<0.01) were observed three days after left EC‐DBS (Figure 4b). The abovementioned significantly changed connections showed no difference between the two groups on Day 28 (Figure S3, all *p* > 0.05). Furthermore, the causal influence flows in the above‐changed connections were also tested using GCA. On day 3, the left PFC exerted a significant influence on the EC (*t* = 4.274, *p* < 0.01) and the DG (*t* = 3.01, *p* < 0.05) as well as an influence from the EC to the DG (*t* = 2.36, *p* < 0.05) in the EC‐DBS group (Figure 4c). In the sham‐DBS group, these influences were not statistically significant (all *p* > 0.05).

### 3.4 Episodic‐like memory performance following EC‐DBS

**FIGURE 5:**
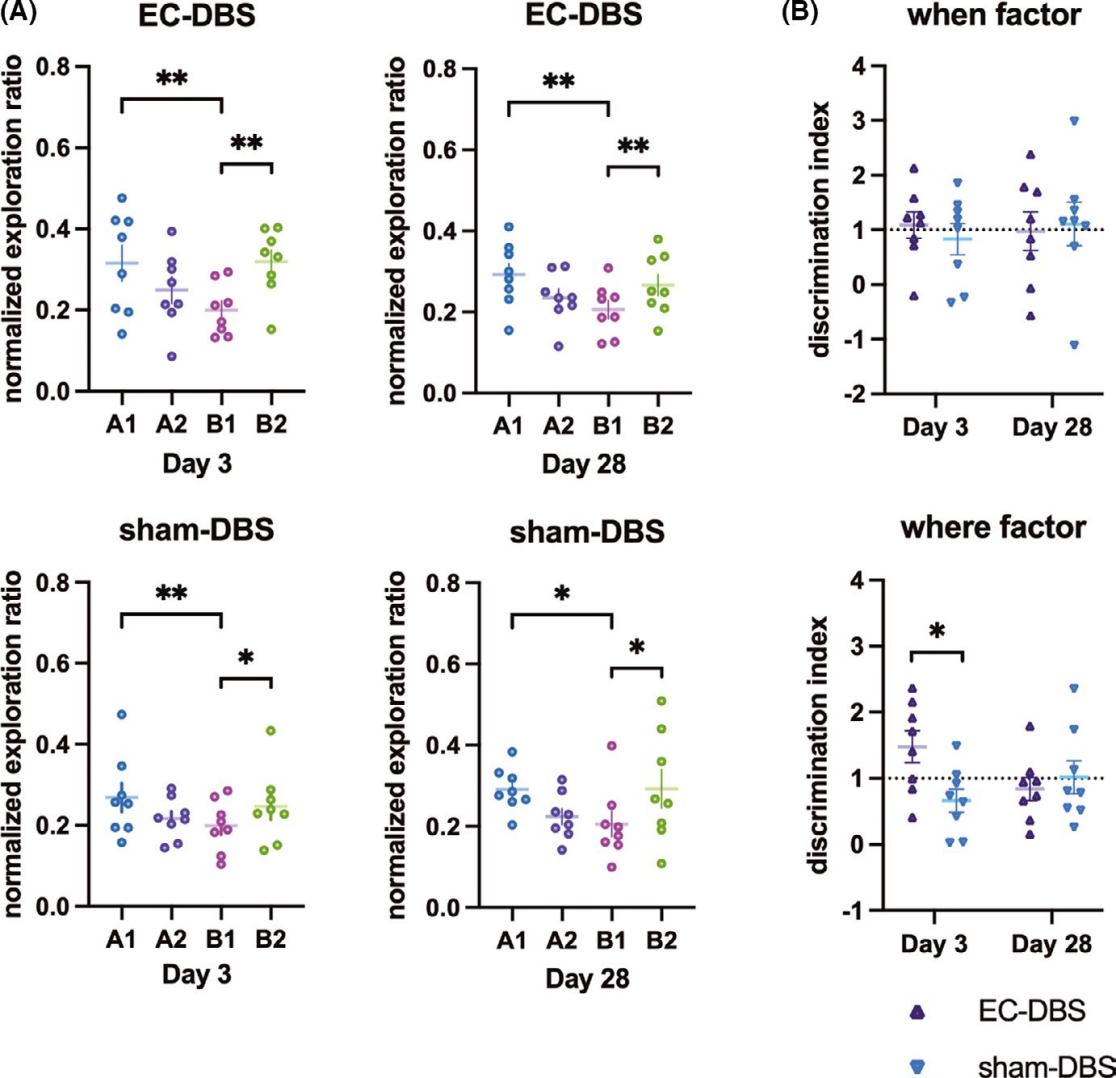
Behavior performance of the animals 3 days and 28 days after stimulation. Normalized exploratory ratio per object in the episodic‐like memory test and the discrimination index (where and when factors) for each group in each test day. Data are represented as mean ± S.E. of all points. *p < 0.05, **p < 0.01

A similar exploration pattern was revealed in both EC‐DBS and sham‐DBS groups in different time points (A1>B1 and B2>B1) (Figure 5a), and the statistic details were shown in Table S1. There was no exploration ratio difference between the EC‐DBS and sham‐DBS groups on Day 3 and Day 28 (all *p *> 0.05, Figure S4). Additionally, EC‐DBS increased the “where” factor as compared to sham‐DBS (F_1,14_ = 6.11, *p* < 0.05), and posttest showed an increased ratio in the EC‐DBS group on Day 3 (*t* = 2.70, *p* < 0.05, Figure 5b). There was no difference in the “when” factor between different groups and different time points (Figure 5b).

## 4 DISCUSSION

Low‐frequency fluctuations in functional networks measured by resting‐state fMRI are thought to reflect neural baseline activity of the brain.^31^, ^34^ Our study showed that 1 h of EC‐DBS temporarily increased the power spectra in the prefrontal and hippocampal‐related networks between 0.01–0.08 Hz, indicating activation in these two networks (Figure 2). Further analysis revealed increased functional connectivity within both the prefrontal and hippocampal‐related networks induced by EC‐DBS, including the orbitofrontal cortex in the prefrontal‐related network and the cingulate cortex in the hippocampal‐related network (Figure 3). In addition, EC‐DBS also exhibited increased connectivity between the hippocampal‐related network and the caudate putamen, a key region in the basal ganglia that is generally accepted to participate in motor control and procedural memory.^35^ Moreover, it is interesting to find that both networks showed increased connectivity with the EC after EC‐DBS. In previous studies, EC‐DBS facilitated an electrophysiological interaction in the hippocampal‐EC network in humans as well as a widespread pattern of BOLD responses in rats, including the PFC, hippocampus and striatum.^7^, ^24^ Similarly, in the present study, we further observed increased functional connectivity among the EC, prefrontal‐ and hippocampal‐related networks in the resting state in a short period after acute EC stimulation, suggesting multinetwork modulation induced by EC‐DBS.

To further observe the modulation effects on each side, the functional connectivity among the ROIs, including the bilateral EC, PFC and hippocampus, was also analyzed. Since subregions of the hippocampus were reported to play different roles in memory function,^36^ the bilateral CA1, CA3 and DG were chosen instead of the hippocampus in the ROI‐based analysis. In our results, increased functional connectivity was mainly revealed on the ipsilateral side of EC‐DBS, and the DG is an important hub region closely related to both the EC and the PFC. Further causal analysis demonstrated that the DG received information flow from both the EC and the PFC (Figure 4). Previous animal studies showed that acute EC‐DBS‐induced DG neurogenesis 3 days after stimulation in healthy animal brains.^11^, ^14^, ^37^ In addition, Liu et al. also reported that ventromedial PFC‐DBS upregulated neurogenesis‐associated genes in the DG along with enhanced HC cell proliferation.^38^ Thus, it is reasonable that the DG functional changes caused by EC‐DBS not only depended on the DG‐EC interaction but also included other hippocampal‐cortex connections. Our results provide neuroimaging evidence for this speculation.

Based on previous findings, DG neurogenesis‐related memory enhancement usually occurs several weeks after EC‐DBS.^11^, ^14^ However, human studies have shown a short‐term memory improvement effect after EC stimulation.^7^, ^8^ One possible explanation is that the choice of behavior tests in human and animal studies was different. Short‐period (within hours) virtual reality tasks were performed in human studies and concentrated on episodic memory and executive function, which are the main clinical impairment in Alzheimer's disease and Parkinson's disease dementia patients.^39^, ^40^ Whereas animal studies mostly used relatively long‐period (3–6 days) tasks focusing on spatial memory, such as the water maze test.^9^, ^11^, ^14^, ^37^ Here, an episodic‐like memory test model was chosen, and the whole test was only lasting a few hours. In addition, to ensure efficacy, bilateral EC stimulation was used in the behavioral testing group. Animals in our results showed normal performance similarly to previous reports (A1>B1and B2>B1).^25^, ^28^ Moreover, EC‐DBS increased the “where” factors on day 3, indicating that acute EC‐DBS could induce a short‐period episodic memory enhancement, especially in spatial discrimination. It's also consistent with previous findings in human studies.^7^, ^8^ Meanwhile, our results showed no behavioral difference between the EC‐DBS and sham‐DBS on day 28. However, these negative results should be cautious to conclude. There are two possible reasons: (1) healthy animals were used in the present study, the memory enhancement effect was not easy to observe because their memory performance was normal; (2) although DBS‐induced better memory performance has been revealed 3–4 weeks after stimulation in some research,^9^, ^38^, ^41^ there were other studies chose 6–8 weeks (to make neurons sufficiently mature) to detect the neurogenesis‐related memory enhancement.^11^, ^13^, ^14^

The acute episodic‐like memory enhancement also suggested that EC‐DBS induces other acute neuromodulation effects beyond neurogenesis, for instance, by increasing the functional connectivity among memory‐related networks, facilitating the coactivation of the important brain regions associated with memory encoding and consolidation. Previous studies in patients with epilepsy reported that EC‐DBS resulted in positive deflections of memory encoding‐related potentials and a theta‐phase resetting in the HC.^7^, ^21^ Animal studies also provide electrophysiological evidence that DBS‐induced long‐term potentiation, a model of synaptic plasticity that is assumed to underlie memory formation, in the hippocampus and sustained for 5–7 days.^42^, ^43^ Other animal research further reported that theta‐gamma phase coupling between the EC and CA1/CA3 could affect information transfer across the regions, and gamma coherence between the CA1 and CA3 could increase memory encoding.^20^ In addition to long‐term potentiation, increased serotonin, acetylcholine and glutamate levels in the hippocampus are also suggested to be involved in the effects of DBS on memory.^44^, ^45^ Besides the undoubtable role of the EC‐hippocampus interaction in memory acquisition, a recent study also emphasized that during memory consolidation, additional synaptic reinforcement takes place within the cortical network, which could change the connectivity of the EC with cortical regions other than the hippocampus.^23^ Krautwald et al. revealed that EC stimulation in rats caused stronger BOLD responses in the PFC compared with the hippocampus, suggesting that the beneficial effects of EC‐DBS might depend on PFC function.^24^ Interestingly, our results also demonstrated an acute EC‐DBS effect on increased resting‐state functional connectivity between the EC and the PFC, the PFC and CA1, and the CA1 and CA3 (Figure 4). Moreover, this network change effect was reversible, which was only observed 3 days after stimulation and recovered to the control level 4 weeks later, indicating that the short‐term and long‐term effects of EC‐DBS might rely on different brain network modulation mechanisms.

In the present study, two time points were chosen to observe the hippocampal‐cortex network and behavioral modulation effect induced by EC‐DBS. Although reversible activation, functional connectivity and behavioral changes were observed, to make the design more rigorous, tests before the stimulation could ensure there is no difference in baseline between the two groups, and more time points after EC‐DBS would provide more details. Additionally, left‐side EC‐DBS was used in our study to detect the resting state changes in both sides of the brain, design of random unilateral stimulation and flipping the direction of the images to one side in later data processing would make the conclusion more robust. Furthermore, to protect the integrity of brain tissue as much as possible, we chose a healthy animal model rather than a diseased model made by stereotactic surgery, however, neuroimaging studies combining behavioral tests in cognitively impaired genetically modified animal models or patients are still needed in future studies. Besides fMRI, PET and electrophysiological methods have been widely used in DBS mechanism studies.^46^, ^47^ Multimodal imaging‐electrophysiological methods in future research would provide high‐temporal resolution evidence of the dynamic brain network responses to EC‐DBS.

## 5 CONCLUSION

Our results showed that acute EC‐DBS‐induced short‐term resting‐state activation in the prefrontal‐ and hippocampal‐related networks as well as increased functional connectivity among multiple brain regions, including the PFC, EC, CA1, CA3 and DG. An obvious information flow was further detected both from the PFC and the EC to the DG. Additionally, acute EC‐DBS also showed a short‐period episodic‐like memory enhancement effect, especially in spatial discrimination. Consequently, EC‐DBS caused a reversible modulation effect on resting‐state functional connectivity in the hippocampal‐cortex network and episodic‐like memory enhancement.

## CONFLICT OF INTEREST

None.

## AUTHOR CONTRIBUTIONS

YJ and DFL designed this research. YJ, DFL, XZ, HGL, and CZ performed the experiments. YJ and DFL participated in data analysis. YJ, DFL, and JGZ wrote the paper. All authors contributed substantially to this research.

## Supporting information

Supplementary Material

## Data Availability

The data that support the findings of this study are available from the corresponding author upon reasonable request.
