# Supplementary material for: Modulation of the rat hippocampal‐cortex network and episodic‐like memory performance following entorhinal cortex stimulation
Source: CNS Neurosci Ther. 2021 Dec 28;28(3):448–57. doi: 10.1111/cns.13795 (PMC8841309; doi:10.1111/cns.13795)
Supplement: Supplementary file 1 — Supplementary Material [file CNS-28-448-s001.docx]

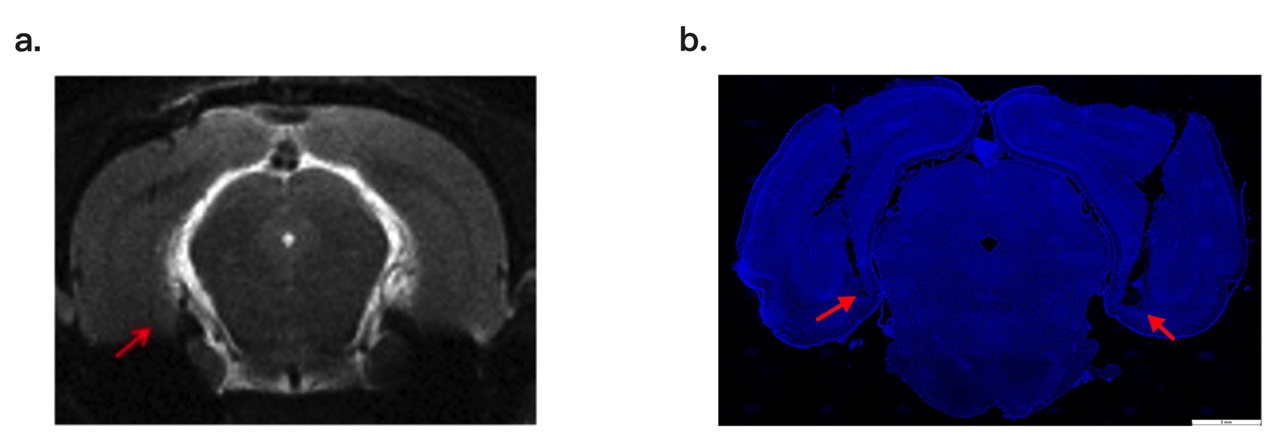


**Figure S1.** Trajectory of the DBS electrodes in T2-weighted MRI for unilateral stimulation animals (a) and in histological staining for bilateral stimulation animals (b).


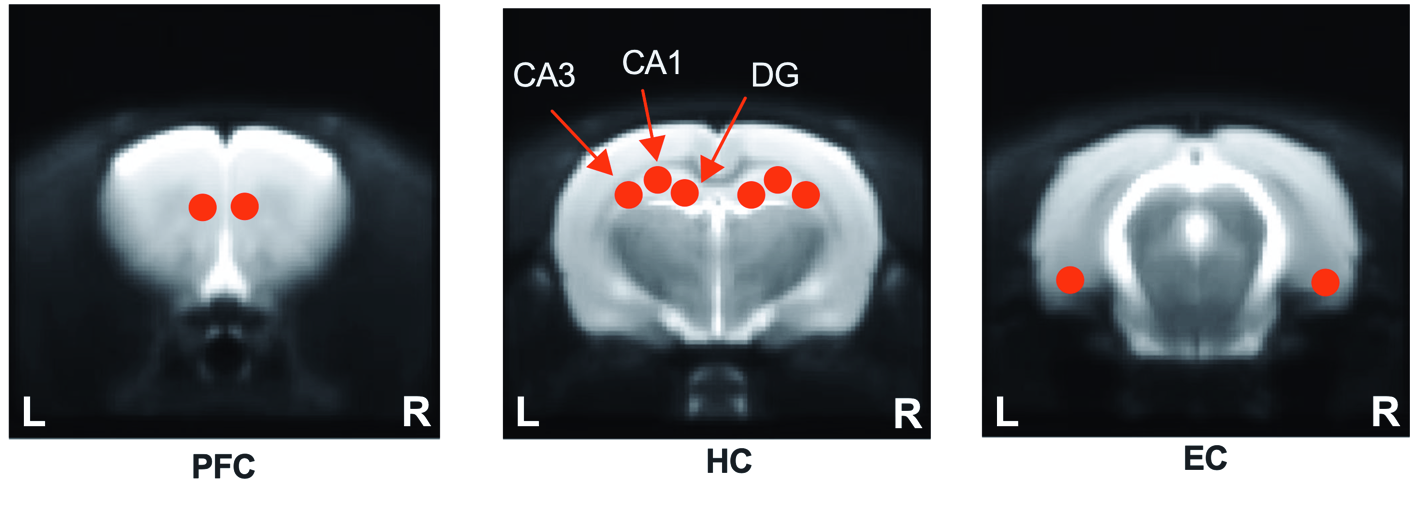


**Figure S2.** Locations of the ROIs in seed-based functional connectivity processing.


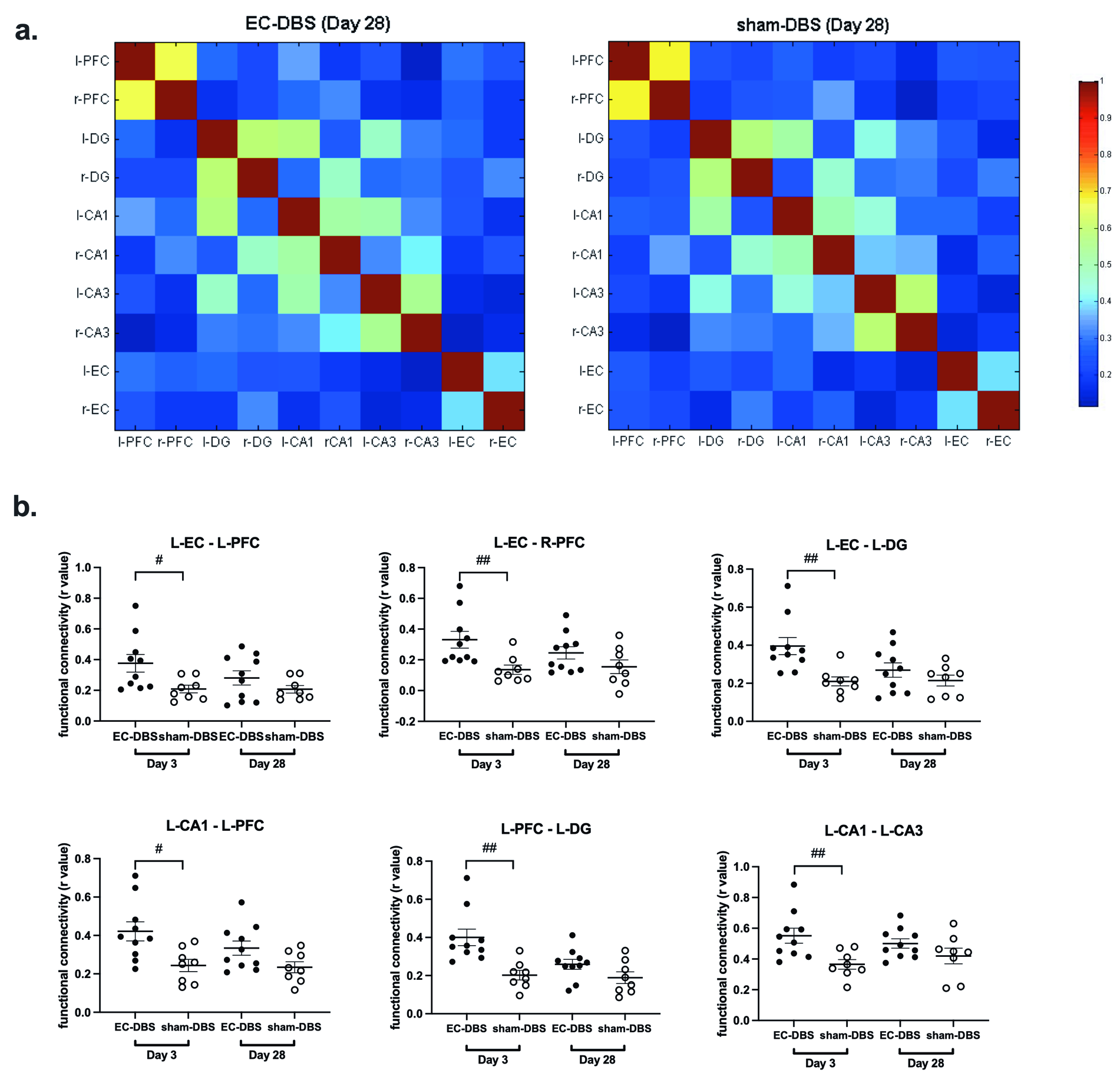


**Figure S3. a.** Functional connectivity among brain regions, including the left and right PFC, DG, CA1, CA3 and EC in the EC-DBS and sham-DBS group 28 days after stimulation. Color bar indicates mean r values. **b.** EC-DBS induced functional connectivity changes. # *P*<0.05, ## *P*<0.01. Data represented as mean ± S.E. of all points.


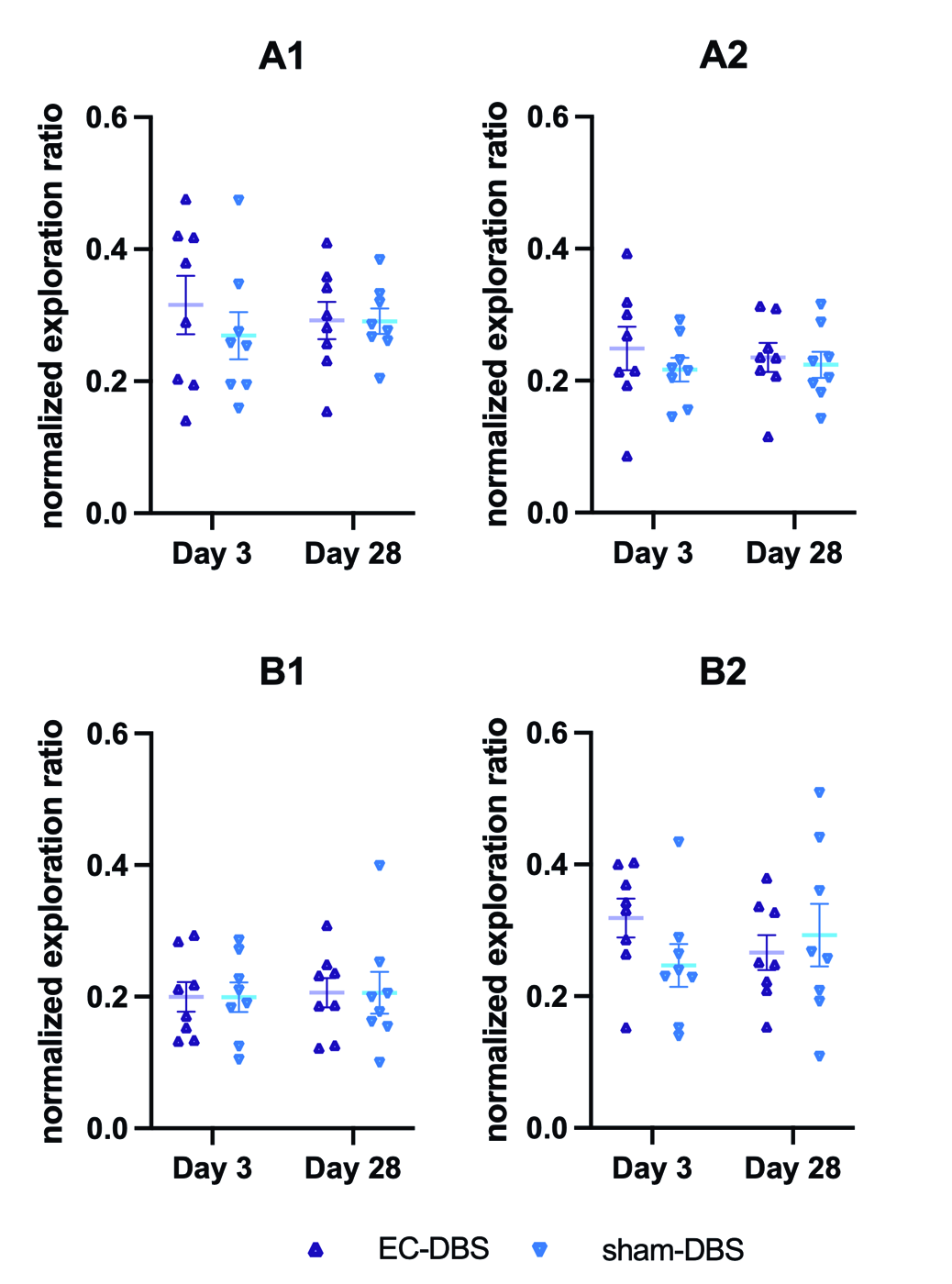


**Figure S4. Exploration ratio of each object in episodic-like memory test.** There was no exploration ratio difference between the EC-DBS and sham-DBS groups on Day 3 and Day 28 (all *P*>0.05). Two-way ANOVA with multiple comparisons test was used, threshold was set to *P*<0.05, FDR corrected. Data represented as mean ± S.E. of all points.

**Table S1 The statistic details of the episodic-like memory test.**

|  |  | **one-way ANOVA** | **A1 > B1** | **B2>B1** |
| --- | --- | --- | --- | --- |
| **EC-DBS** | day 3 | F_3,21_=8.59, *P*<0.001 | *t* = 4.19, *P*<0.01 | *t* = 4.30, *P*<0.01 |
|  | day 28 | F_3,21_=7.11, *P*<0.01 | *t* = 4.34, *P*<0.01 | *t* = 3.04, *P*<0.01 |
| **sham-DBS** | day 3 | F_3,21_=4.29, *P*<0.05 | *t* = 3.29, *P*<0.01 | *t* = 2.24, *P*<0.05 |
|  | day 28 | F_3,21_=4.28, *P*<0.05 | *t* = 2.77, *P*<0.05 | *t* = 2.82, *P*<0.05 |
